# Participant perspectives of a home-based palliative approach for people with severe multiple sclerosis: A qualitative study

**DOI:** 10.1371/journal.pone.0200532

**Published:** 2018-07-12

**Authors:** Ambra Mara Giovannetti, Claudia Borreani, Elisabetta Bianchi, Andrea Giordano, Sabina Cilia, Susanna Cipollari, Ilaria Rossi, Claudia Cavallaro, Valentina Torri Clerici, Edoardo Rossetti, Maria Consiglia Stefanelli, Amadio Totis, Angelo Pappalardo, Gina Occhipinti, Paolo Confalonieri, Simone Veronese, Maria Grazia Grasso, Francesco Patti, Paola Zaratin, Mario Alberto Battaglia, Alessandra Solari, on behalf of the PeNSAMI project

**Affiliations:** 1 Unit of Neuroepidemiology, Foundation IRCCS Neurological Institute C. Besta, Milan, Italy; 2 Department of Neuroimmunology and Neuromuscular Diseases, Foundation IRCCS Neurological Institute C. Besta, Milan, Italy; 3 Unit of Clinical Psychology, Foundation IRCCS Istituto Nazionale per la Cura dei Tumori, Milan, Italy; 4 MS Centre, Neurology Clinic, University Hospital Policlinico Vittorio Emanuele, Catania, Italy; 5 Multiple Sclerosis Unit, Foundation IRCCS S. Lucia, Rome, Italy; 6 ANTEA Charitable Foundation, Rome, Italy; 7 FARO Charitable Foundation, Turin, Italy; 8 Scientific Research Area, Italian Multiple Sclerosis Foundation (FISM), Genoa, Italy; 9 Department of Life Sciences, University of Siena, Siena, Italy; University of Birmingham, UNITED KINGDOM

## Abstract

**Background:**

We performed a qualitative study to investigate the experiences of participants in a multicentre randomized controlled trial on a home-based palliative approach (HPA) for adults with severe multiple sclerosis (MS) and their caregivers. Our aim was to explore the strengths and challenges of the intervention, and circumstances that may have influenced its efficacy.

**Methods:**

Participants to the qualitative study were the patients, their caregivers, patient referring physicians, and the teams who delivered the HPA intervention. We performed semi-structured one-on-one interviews with 12 patients and 15 informal caregivers chosen using a maximum variation strategy, two focus group meetings with patient referring physicians (4 participants each), and one with the HPA teams (9 participants).

**Results:**

From data analysis (framework method) 38 sub-categories emerged, which were grouped into 10 categories and 3 themes: 'expectations,' 'met and unmet needs', and 'barriers’. Intervention benefits were improved control of symptoms and reduced sense of isolation of the patient-caregiver dyads. Limitations were: factors related to experimental design (difficulty of dyads in identifying examiner and team roles, additional burden for caregivers); team issues (insufficient team building /supervision, competing priorities); limitations of the intervention itself (insufficient length, lack of rehabilitation input); and external factors (resource limitations, under-responsive services/professionals). The referring physician focus groups provided little experiential data.

**Conclusions:**

The HPA reduced patient symptoms and sense of isolation in patients and caregivers. The indirect role of the HPA teams, and insufficient length of the intervention were key limitations. The experimental design imposed additional burdens on the dyads. Key barriers were the paucity of available services, the demanding administrative procedures, and lack of networking facilities. These findings suggest that two major requirements are necessary for home palliative care to be effective in this patient population: HPA teams well-connected with MS rehabilitation services, and care delivered over the long-term, with variable intensity.

**Trial registration:**

Current Controlled Trials ISRCTN73082124 (Registered 19/06/2014).

## Introduction

Multiple sclerosis (MS) is a chronic neurological disease with unpredictable course. Around 15% of MS patients have a primary progressive course, and a further 35% develop secondary progressive disease after a variable period with a relapsing-remitting disease [1]. Patients may experience a wide range of symptoms including reduced mobility, compromised sphincter control, impaired cognition, difficulties with swallowing and speech, and pain and sensory disturbances [2–4]. Aspiration pneumonia, urinary tract infections, complications of falls and fractures, and sepsis secondary to pressure ulcers are not rare and may lead to death in people with severe MS [5]. No treatments are able to reliably delay or prevent further clinical worsening.

A qualitative study that explored the needs of people with severe MS found that disability and poor quality of life (QOL) were the main worries, while death was not a concern [6]. Improving QOL is the main aim of palliative care. However, the few trials assessing palliative care in people with MS have not provided univocal findings: the UK randomized controlled trial (RCT) found no effect of a home specialist palliative service on the primary outcome (emotional, psychological, and spiritual needs of MS patients) while some symptoms improved and informal caregiver burden was reduced [7,8]; the Italian Neurology-Palliative care RCT (Ne-Pal) found an improvement of patient QOL and some symptoms, but no effect on caregiver burden [9].

The PeNSAMI (Palliative Network for Severely Affected Adults with Multiple Sclerosis in Italy) trial, the most recently published study, was a two-arm parallel, examiner-blind RCT that assessed a home palliative approach (HPA) in 50 patient-caregiver dyads versus usual care in 26 dyads. Participant characteristics and the intervention are summarized in S1 Appendix; the full trial protocol and results are reported elsewhere [10,11]. Although the PeNSAMI intervention was carefully planned with the direct participation of key stakeholders [6], it was less effective than anticipated: while symptom burden was reduced, the reduction was later than expected, and changes in QOL and other patient and caregiver outcomes did not differ compared to usual care [11].

The present qualitative study, nested within the PeNSAMI trial, assessed the experiences and perspectives of: patients with severe MS and informal caregivers who received the intervention; the teams that delivered the intervention; and the patient referring physicians. The qualitative design was used to ‘augment’ the RCT findings: qualitative data collection and analysis took place after the intervention, to help explain the (quantitative) results, explore how the intervention worked in practice, identify its strengths and limitations, and contextual factors that may have influenced its implementation.

## Methods

Our referral framework was the mixed methods approach which, by integrating the quantitative findings with the qualitative findings, aims to provide a more comprehensive picture of the intervention than either method can do [12]. More specifically, we used an explanatory sequential approach which involved two phases: an initial quantitative phase (the PeNSAMI trial), followed by a qualitative data collection phase (the present study) aimed to better understand, via the personal experiences of trial participants, the trial findings.

The framework method of analysis was chosen because it is particularly suitable to explore and describe the subjective experience of the different players (patients, caregivers and health providers) involved in the implementation of the home care intervention [13]. Our aim was not to generate a theory but to capture the different aspects and perspectives of the phenomenon under investigation through an inductive approach. Furthermore this is a dynamic method which allows change, addition or amendment throughout the analysis process [14].

For the patients and the caregivers the home-based personal semi-structured interviews were considered most appropriate to limit interview burden and hopefully make it easier for participants to express their feelings, and recount their experiences of the intervention. For the teams and the patient referring physicians we chose the focus group meetings as they promote interaction and exchange of ideas.

### Interviews and focus group meetings

The PeNSAMI protocol [10] specified that the qualitative study should proceed by holding (a) personal semi-structured interviews with patients who received the intervention and their caregivers, and (b) three focus group meetings (one for each center) for the referring physicians of patients who received the intervention. As experiential data were negligible from analysis of two referring physician meetings, we decided not to hold the third. An additional focus group meeting involving all the three intervention teams was also held (not contemplated in study protocol). The consolidated criteria for reporting qualitative research (COREQ) guided the reporting of findings [15,16]. Adherence to the COREQ checklist is documented in S1 Checklist. Guides to conducting the interviews and focus group meetings (S2 Appendix) were developed by the PeNSAMI qualitative panel, with input from the steering committee. After piloting in two dyads, minor changes were made to the structure and order of the questions in the interview guide.

#### Interviews

Patients and caregivers were interviewed separately at patient homes. The interviews took place within six months of trial completion, and lasted no more than an hour (patients) or an hour-and-a-half (caregivers).

A minimum of 12 patient and 12 caregiver interviews was planned. Data from each set of interviews were analyzed immediately and used to decide the characteristics of the next interviewee dyad, revise the interview guide (if necessary), and indicate when interviewing should cease because of data saturation [17].

The interviews were conducted in Milan by a psychologist experienced in qualitative research (EB), and in Rome and Catania by specially trained psychologists (SCip and SC, respectively). Neither patients nor caregivers had met the interviewers previously. A purposive sampling technique was used to select the participants (at least four dyads from each center) who obtained the greatest and least benefit from the intervention (in terms of primary outcomes). At least two caregivers of patients with severe cognitive compromise (patients not interviewed) were also selected.

Before starting, interviewees were informed of study aims and requirements, and provided written consent, as required by the Helsinki Declaration and EU Good Clinical Practice guidelines. The interviewer then explained that the aim of the interview was to obtain participant feedback on experience of the PeNSAMI trial and stressed that positive and negative experiences of, and feelings about, the intervention were welcome. Participants were assured that the interviews were confidential, and that the audio recordings and subsequent transcripts would be fully anonymized. The interviewer then posed each question in turn, neutrally (so as to not suggest any particular reply) and in an open-ended fashion (to allow many possible replies). As each question was discussed, follow-up questions clarified and explored participant responses. Participants were also encouraged to elaborate on any pertinent themes or views that emerged. The interviewer also noted any potentially informative non-verbal gestures. At the end of the interview, the interviewer verbally summarized the key points and asked the participant if the summary was full and correct.

#### Focus group meetings

All participants provided written informed consent prior to participating. The meetings were audio-recorded and transcribed verbatim. All were conducted by a single facilitator (EB, not acquainted with any participant except the psychologist of the team of Milan), whose job was to engage all participants, promote exchange, moderate conflicts, ensure that all pre-specified topics were adequately covered, and allow exploration of any pertinent issues that arose. She first explained the purpose of the meeting and asked participants to introduce themselves. She then introduced each topic in turn, in an open-ended fashion. At any point the facilitator could probe for further information and ask follow-up questions to stimulate further discussion. After all pre-specified topics had been fully discussed, the facilitator summarized the main points, and asked for further feedback and whether all concerns had been fully aired. The co-moderator (AS) took notes and oversaw the audio recording. Subsequently, the facilitator produced a report from the audio recordings/transcript and her field notes, which was submitted to participants for review (respondent validation).

### Ethics statement

The protocol and consent procedures were approved by the Ethics Committee of: Foundation IRCCS Neurological Institute C. Besta, Milan (refs. no. 06, 5 March 2014; 11, 3 September 2014); Foundation IRCCS S. Lucia, Rome (ref. no. CE/OSS.27, 16 October 2014); University Hospital Policlinico Vittorio Emanuele, Catania (ref. no. 38776, 2 October 2014).

### Analysis

The methods of framework analysis were applied to the data. Framework analysis uses an inductive approach to identify the themes and categories that emerged from the interviews and meetings [17–19]. Two psychologists (EB and CB) experienced in qualitative research and not involved in MS patient care, analysed the transcripts in six steps, independently (steps 1–5) and jointly (step 6): (1) In a given transcript the researcher identified all propositions thought significant, without considering their relation to other parts of the transcript, and added comments. (2) Comments were subsequently expanded and related to other points that arose. (3) Relations between comments were established by re-ordering and re-grouping by sub-categories. (4) The sub-categories considered relevant were ordered hierarchically into categories, moving from the specific to the general. (5) The analyses of each transcript were then compared with each other to identify common and one-off sub-categories. (6) The analyses produced by each researcher were compared, and a consensus arrived at. The six phases of the analysis are exemplified in S3 Appendix.

A joint report of interview and focus group meeting findings was produced. In this report − and the present paper − the categories identified were compared with those identified in the qualitative study used to inform the development of the PeNSAMI intervention [6]. Where appropriate, the previous category names were used to facilitate comparison.

## Results

### Participants and settings

Between October 2015 and April 2016, 27 interviews of mean duration 28 minutes (range 11–60) were conducted, 12 with patients and 15 with caregivers (Table 1). Two of the contacted dyads refused to participate: one because unavailable during the period allocated for interviews, the other because the carer was too occupied with caregiving.

**Table 1:** Characteristics of participants of personal semi-structured interviews.

	Persons with severe MS (N = 12)	Caregivers (N = 15)
	**N (%)**	
Women	7 (58)	9 (60)
Age (years)^1^	58 (43–78)	65 (23–81)
Education: Primary (5–8 years)	6 (50)	5 (34)
Secondary (12–13 years)	4 (33)	4 (26)
College/University (14+ years)	2 (17)	6 (40)
Occupation: Employed/student	1 (8)	7 (47)
Retired (for age)	2 (17)	7 (47)
Housewife	0	1 (6)
Retired (for disability)	9 (75)	0
EDSS score^1^	8.5 (8.0–9.5)	
Age at MS diagnosis (years)^1^	38 (26–66)	
Relation to patient: Spouse/partner		6 (40)
Parent		3 (20)
Son/daughter		3 (20)
Other relative		3 (20)
POS-S-MS outcome^2^: Improved	6 (50)	
Not improved/worsened	6 (50)	
Center: Milan	5 (42)	5 (33)
Rome	3 (25)	5 (33)
Catania	4 (33)	5 (34)

EDSS is Expanded Disability Status Scale; MS is multiple sclerosis; POS-S-MS is Palliative Care Outcome Scale-Symptoms-Multiple Sclerosis.

1. Median (min, max).

2. Three additional patients (2 improved) were not interviewed (severe cognitive impairment) and outcome was assessed by the caregiver version of POS-S-MS.

The referring physician focus group meetings took place in June (Foundation IRCCS Neurological Institute C. Besta, Milan, 60 minutes) and July (Foundation IRCCS S. Lucia, Rome, 90 minutes) 2016. Of the 34 eligible physicians, 21 (61.7%) refused to participate. Reasons for refusal were work commitments (n = 19) and holidays (n = 2). Five agreed to participate but did not show up for unscheduled work commitment (n = 3), family commitment (n = 1), and oversight (n = 1). The characteristics of the eight participants (4 per meeting) are shown in Table 2.

**Table 2:** Characteristics of focus group meeting participants.

	Referring physicians (N = 8)	HPA team members (N = 9)
	**N (%)**	
Women	4 (50)	6 (67)
Age (years)^1^	56 (36–66)	47 (31–61)
Profession: Family doctor	2 (25)	
Neurologist^2^	6 (75)	2 (22)
Nurse		3 (34)
Psychologist		2 (22)
Social worker		2 (22)
Number of focus group meetings	2	1

HPA, home palliative approach.

1. Median (min-max).

2. One team neurologist was also a physiatrist.

The HPA team meeting was held at the Foundation IRCCS Neurological Institute C. Besta, Milan, in October 2016 and lasted 120 minutes. Of the 12 eligible, 6 participated in person, 2 via videoconference (GO, Catania team social worker; AMG, Milan team psychologist) and 1 via audioconference (MCS, Catania team nurse). Three could not attend, 2 for work commitments (physician and social worker of Rome team) and the other for personal reasons (Catania team psychologist).

### Qualitative findings

The two referring physician focus group meetings produced little experiential data and their findings are presented separately. From the patient and carer interviews, and HPA team focus group meeting, 38 sub-categories emerged which were grouped into 10 categories and three themes: ‘expectations,’ ‘met and unmet needs’, and ‘barriers.’ The findings are presented by theme, with quotations to illustrate their derivation. The provenance of quotations is indicated as patient (P), carer (C), or team member (T), with other information included as appropriate (sex, age, Expanded Disability Status Scale [EDSS] score [20], relation to patient, profession, and center).

#### Expectations about the study

It was also possible to distinguish between attitudes mediating expectations and specific expectations contents (Table 3).

**Table 3:** The two expectation categories (columns) and eight sub-categories (rows) reported by participants.

Category	Attitudes mediating expectation	Expectation contents
Sub-category	• Curiosity (P, C) • Skepticism* (P, C) • Disillusionment* (P)	Disease management • Sharing disease experience (P, C, T) • Receiving expert opinion (C) • Receiving practical and tangible support (C, T) • Clinical improvement (P, C) Research and knowledge • Increasing scientific knowledge (P, C, T)

P, patients; C, caregivers; T, teams

* identifies negative attitudes.

As regards attitudes mediating expectations, patients and caregivers expressed curiosity about the intervention. *“No*, *more than expectations*, *I would say curiosity*. *I wanted to understand what this proposal was really about*. *I was curious about palliative care in general*.*”* [C: husband, 65 years, Rome]. *“I am a curious person*, *so I agreed immediately*. *I didn’t have any questions*, *I didn’t want to waste time*.*”* [P: man, 43 years, EDSS8.5, Catania]. Some participants expressed skepticism and disillusionment. *"I didn’t have high expectations*. *I thought we had nothing to lose […] but I wasn’t hoping for anything*.*”* [C: daughter, 42 years, Milan]. *“No*, *no*. *No expectations*. *I judge things as they happen*. *I'm pretty disillusioned [*…*] I wasn’t expecting immediate results*.*"* [P: woman, 58 years, EDSS 8.5, Rome]. *“I didn’t expect much more*, *I didn’t think they would change my life*. *Not at all*.*”* [C: wife, 73 years, Milan]

Expectations contents concerned disease management and research and knowledge. As regards disease management, four sub-categories arose: sharing disease experience; receiving expert opinion; receiving practical support; and clinical improvement. All three groups expected the project to be a good opportunity to share disease experiences: *"I saw it as an opportunity to talk about my experience"* [P: woman, 47 years, EDSS 8.0, Milan]; “*If he can be in company and speak about himself*, *bring it on*!” [C: wife, 67 years, Catania]; *“We expected to know these families*, *to share moments with them”* [Male nurse, Milan team]

Caregivers expected to meet professionals who would provide practical advice and concrete support. *"I hoped to get advice on what to do […] from someone who could appraise the situation at home*.*"* [C: brother, 48 years, Rome]. HPA teams had similar expectations: *"I figured that we could free ourselves from our defined role [indirect intervention] and be more hands on*, *more helpful to the dyads*.*”* [Female social worker, Catania team].

Dyads expected the intervention to improve patient health. *“I hoped it would improve all this [the disease]*, *be something useful*.*”* [P: man, 78 years, EDSS 8.5, Rome]. *“I expected my son to improve”* [C: mother, 75 years, Catania].

With regard to research and knowledge, all participants expected the project to increase knowledge about living with and caring for people with advanced MS, and thus guide future clinical practice. *“I thought [the study] would help them better understand the disease”* [P: woman, 76 years, EDSS 9.0, Milan].*“They explained that this study aimed to better understand patient needs”* [C: wife, 73 years, Milan].*“We thought it would lead to deeper understanding of patient issues*.*”* [Male social worker, Milan team].

#### Met and unmet needs

Needs that had and had not been met by the intervention were grouped into disease management, and psychological and social issues (Table 4).

**Table 4:** The two needs categories (columns) and 16 sub-categories(rows) reported by participants.

Category	Disease management		Psychological and social issues	
	*Met need*	*Unmet need*	*Met need*	*Unmet need*
Sub-category	• Symptom management (P, C, T) • Aids & medical devices (C, T) • Point of reference (C, T)	• Home health care (P, C, T) • Qualified MS health professionals & a case manager (P, C, T) • Physiotherapy (P, C, T)	• Emotional support (P, C, T) • Reassurance (C, T) • Communication (P, C) • Information (P, C, T) • Administrative issues (P, C, T)	• Social integration (P, C, T) • To help others (P) • Psychological support (P, C, T) • Management of family problems (T) • Reduction of caregiver burden (P, C, T)

P, patients; C, caregivers; T, teams.

For disease management, improvements were reported in terms of symptoms management, aids and medical devices, and presence of a reference point. All three parties reported that the intervention was effective in reducing pain and other symptoms. *“I’m taking that drug and I must say the pain has stopped*. *She gave it to me to try*, *and I’ve had no pain since I started*.*”* [P: woman, 67 years, EDSS 8.0, Milan]. *“She complains less than before*, *especially about night pain*. *She still has pain*, *but it’s not as strong as before*.*"*[C: husband, 67 years, Milan]. *“Patients’ requests very often concerned medical issues*, *such as pain or other symptoms… I had to intervene*!*”* [Male neurologist, Catania team]. Caregivers and team members reported that the intervention resulted in improved use of devices/procedures like percutaneous endoscopic gastrostomy (PEG).*"With PEG it was fundamental to educate our patients about how to manage and clean the device*.*”* [Male physician, Catania].*“They helped us find a more suitable wheelchair…”* [C: sister, 56 years, Catania]. Participants also reported presence of a reference point as an improvement. *“If I had a neurological emergency*, I’d[now] call Dr *** and ask for advice” [C: husband, 61 years, Milan]. *“I’m still in touch with many of them*. *We exchange emails*, *phone each other…”* [Female social worker, Catania].

Findings on psychological and social issues were grouped into emotional support, reassurance, communication, information, and administrative issues. All parties recognized that the dyad derived emotional support from interaction with the team. Patients and caregivers in particular perceived the interaction as an opportunity to stay in contact with their feelings and to express them. *“I think they did a good job psychologically […] they dug inside me”* [P: man, 55 years, EDSS 8.5, Catania]. *“I just opened up and said what I was thinking*. *It was comforting when they came […] we spoke differently than usual*” [C: husband, 67 years, Milan]. *“We dedicated time talking together during each visit*. *Some of them needed psychological support more than nursing assistance”*[Female nurse, Rome team]. Caregivers and team members also reported a reassurance effect. *“The opportunity to ask them [the team] for suggestions*, *made me feel safer*.” [C: sister, 56 years, Catania]. *“Being there is something really positive for them and it is strictly connected with one of their major needs*: *safety and reassurance”* [Female Social Worker, Catania Team]. Similarly, caregivers and patients reported that interaction with the team was an opportunity to communicate and exchange opinions, a first step to resolving difficulties. *“We solved things because of this dialogue [*…*] I improved simply because of this dialogue with them*.*”* [P: man, 53 years, EDSS 8.5, Catania]. *“Having the opportunity to interact with health professionals with specific competences was worthwhile*.*”* [C: daughter, 42 years, Milan].

The team also provided the dyads with practical information. *“It was beautiful for a patient to receive explanations and many other things”* [P: man, 44 years, EDSS 8.5, Catania]. *“We made some modifications based on nurse’s and social worker’s suggestions”* [C: brother, 48 years, Rome]; *“We suggested how they could get what they were entitled to*, *even though they hardly ever got it in practice*.*”* [Female social worker, Catania]. All parties reported that the intervention had a positive impact on administrative issues. *“They hastened procedures at the local health offices”* [C: brother, 48 years, Rome]. *“Several times the social worker went to city offices with my husband […] to discuss things*. *In short*, *she always went along with him*.*”* [P: woman, 59 years, EDSS 8.5, Rome]. *“We reduced caregiver’s burden by providing them with clear indication about administrative procedures”* [Female Social worker, Catania Team].

Despite these improvements, some needs (same two categories as above) remained unmet. For disease management, all parties complained that home care was lacking. *"It‘s important [for doctors] see us at home […] it gives the possibility of a more meaningful interaction*.*"* [P: man, 55 years, EDSS 8.5, Catania]. *"We always have to go to the doctor*. *[*…*] In our situation a home visit would be more than welcome*!*"* [C: wife, 73 years, Milan]. *"With some treatments*, *compliance is low because no one is monitoring it from week to week*. *Going to the patient’s home can be crucial*.*"* [Female physician, Milan]. It was also noted that qualified health professionals, and a case manager to coordinate health professionals and match dyad needs to available resources, were not available. *"We need experienced MS specialists who visit the patients at home [*…*] the family doctor does not feel competent enough [*…*] we need skilled professionals around the patient*.*"* [C: sister, 56 years, Catania]. *"Yes*. *Coordination is required*.*"* [P: woman, 47 years, EDSS 8.0, Milan]. *"Give me a [single] reference point without having to go through thousands of levels*.*"* [C: mother, 67 years, Milan]. *“These patients need a case manager*, *someone able to integrate all the competencies*, *someone able to notice family needs and to matching them with the resources available*.*”* [Female Neurologist, Milan Team]. All three parties said that more physiotherapy was needed. *“Now what I really need is physio”* [P: woman, 47 years, EDSS 8.0, Milan]. *“We need all kinds of services… she should have more physio*.*”* [C: husband, 61 years, Milan]. *“Sometimes their unmet needs were really concrete*: *more physiotherapy*!*”* [Female Psychologist, Milan Team]

As regards psychological and social needs, all parties found deficits in available resources which limited intervention efficacy. Unmet psychological and social needs were grouped into social integration, helping others, psychological support, management of family problems, and reduction of caregiver burden. Social integration and connection emerged as crucial for all parties. Dyads and teams complained strongly about the lack of means to promote dyad social participation. *“If only I had people at hand to talk to*, *to go out with*.*”* [P: woman, 57 years, EDSS 8.0, Milan]. *“Letting them get out a bit*, *maybe with the family*, *for a day*, *all together*, *relatives included [would be good]*.*”* [C: sister, 56 years, Catania]. *“I wish patients could get out of the house*, *but we haven’t had much success with this; they stay at home with all their problems[…] in short*, *they need some activity”* [Female nurse, Catania].

Some patients expressed a desire to help others, both as a way of being part of the community and as a means of expressing themselves as individuals. *“I am sick but I also want to help others*, *as long as I have the strength to speak [*…*] I’d also like to tell others how I feel*.*”* [P: woman, 47 years, EDSS 8.0, Milan].

The need for psychological support was reported by dyads, and recognized as important by teams. *“They* [the team] *came*, *and asked me*: *What about meeting a psychologist*? *I answered*: *It sounds great to me*!*”* [P: woman, 58 years, EDSS 8.5, Rome]. *"I need to talk to a psychologist*, *I need to communicate*.*"* [C: female partner, 67 years, Catania]. *"Very few of them go to a psychotherapist or get any kind of psychological support*. *Everything is buried*, *swept under the rug*.*"* [Female psychologist, Rome]. Consistent with this, teams reported that some families experienced relational conflicts, for which psychological support was needed. However it was impossible for teams access such support in the public health domain. *“It all came out—conflicts between family members that had degenerated and had a negative impact on patient management*. *[*…*] We couldn’t find any services [to deal with this]*.*"* [Female social worker, Catania].

Reducing caregiver burden was another important issue. Participants reported it was burdensome to be the only carer, and hard to find someone to relieve them. *"Well*, *I need someone to help [my husband]*. *He had a heart attack"* [P: woman, 68 years, EDSS 8.0, Milan]. *"One person is not enough to take care of a patient like this"* [C: sister, 56 years, Catania]. *"I don’t say I don’t need any help*, *although I can manage*. *At times though I really wish I had help*. *There are days when I go well*, *I manage [*…*] here full-time*: *I move her*, *dress her*, *undress her*, *put her to bed*.*"* [C, husband, 67 years, Milan]. *“Caregiver depression and stress is always really high*!*”* [Male Nurse, Milan Team].

#### Barriers

The main barriers (Table 5) reported by dyads and teams, that limited intervention efficacy, were: organizational/structural; experimental design; team issues; and dyad issues.

**Table 5:** The four barrier categories (columns) and 14 sub-categories (rows) reported by participants.

Category	Organization/Structure	Experimental design	Team	Dyad
Sub-category	• Insufficient services (C, T) • Lack of networking facilities (C, T) • Complex administrative procedures (C, T) • Unsuitable housing (P, C)	• Intervention too short (P, C, T) • Burdensome examiner visits & telephone interviews (P, C) • Dyad difficulty in identifying examiner & team roles (P, C, T) • Invasiveness (C) • Hands-off role of team (C, T)	• Lack of other health care professionals (C, T) • Need for more teambuilding (T) • Insufficient supervision of teams (T)	• Difficulty expressing needs (T) • Dysfunctional dyads (T)

P, patients; C, caregivers; T, teams.

Organizational barriers were insufficient services, lack of networking facilities, complex administrative procedures, and structural factors (e.g. unsuitable housing). Inadequacy of services prevented resolution of difficulties even after the team identified them. *"One can be a facilitator when services are in place*, *but when there are no services what can I facilitate*? *In Rome*, *for example*, *many [patients] do not get rehabilitation*, *because of limited mobility*, *and ambulances cost too much"* [Female psychologist, Rome]. *“There remains the problem of lack of services provided by the local health authority [*…*]services are few and badly delivered*.*"*[C: brother, 48 years, Rome].

Lack of networking limited the efficacy of the intervention. *“A better collaboration between services would be greatly appreciated*.*”* [C, daughter, 42 years, Milan]. The family doctor viewed the intervention as an occasion for respite rather than for collaboration and networking. *"You have difficulty in contacting the family doctor*, *and when you finally do*, *he says well*, *there you are*, *one more reason not to involve me; [*…*] they’re good professionals*, *but obviously in this system they have a hard time*.*"* [Male nurse, Milan ]. *"I informed them [family doctors]*. *I sent emails summarizing the study*, *and then we sent them our reports*: *social*, *psychological*, *nurses’ and medical*. *But no answer*.*"* [Female nurse, Catania].

The complexity of administrative procedures emerged as a major burden. *“Better access to services is needed [*…*] you always have to start procedures well in advance”* [C: daughter, 42 years, Milan]. *“We [the team] often experienced the powerlessness of being unable change things because of bureaucratic inertia*.*”* [Female social worker, Catania].

Unsuitable housing sometimes prevented the introduction of devices or adaptations. *“I don’t have the space to install a lifter*.*”* [P: woman, 68 years, EDSS 8.0, Milan]. *“We have concrete difficulties […] we need a special chair like one used in ambulances to transfer patients*. *Every time I go with her to see the doctor [lack of such a chair] makes it always so complicated*.*”* [C: daughter, 44 years, Rome].

With regard to experimental design, four limitations emerged: intervention too short, burdensome examiner visits and telephone interviews, difficulty defining examiner and team roles, indirect (hands-off) role of the team. All three parties reported the need for an intervention lasting more than six months. *"I was hoping it would last longer*.*"* [P: man, 55 years, EDSS 8.5, Catania]. *“The intervention should have been more intense and it should have last longer*!*”* [C: brother, 48 years, Rome]. *“As soon as you start working with the dyad*, *the 6 months intervention ended*! *We had the feeling to have not enough time*!*”* [Female Psychologist, Milan Team].

Both patients and caregivers perceived the assessment as burdensome. *“They asked me things I was not interested in*.*”* [P: woman, 58 years, EDSS 8.5, Rome]. As the intervention addressed the patient-caregiver dyad, the caregiver had to be present during team visits. Furthermore since patient and caregiver outcomes were endpoints (and for patients with severe cognitive compromise caregiver-reported patient outcomes were used) the caregiver also had to be present during the (blinded) examiner visits. *“They wanted me to be present […] getting organized wasn’t easy*. *Then there was never agreement about the date [*…*] they said we lived far away [*…*] I work in the afternoon and have a son*, *a little boy [*…*] these are things to keep in mind*.*"* [C: daughter, 44 years, Rome]. *"They all came separately [team and examiner] but asked the same questions*.*”* [C: daughter, 42 years, Milan]. Participants were confused about the role of the different health professionals (blinded examiners, telephone interviewers, team members). *“I don’t remember whether he was the social worker […]I remember he was a man*, *that’s all I remember*.*”* [P: woman, 77 years, EDSS 9.0, Milan].*“Once two people came [*…*] but I don’t know who they were*. *For us it was all the same*, *I no longer knew who came and who did what*.*”* [C: daughter, 42years, Milan]. *“It was difficult to figure out who was supposed to do what* … *the when*, *the what*, *and the how*.*”* [Male social worker, Milan]. Some dyads reported that the home visits were invasive. *“Familiarity… with strangers coming into your house […] it’s not easy*!*”* [C: daughter, 44 years, Rome].

Caregivers and team members complained about hands-off role of the HPA teams. *“Some more help… something more concrete […] would have been appreciated*.*”* [C: brother, 48 years, Rome]. *“I can’t say it was a great experience*. *They came*, *they listened*, *but they did nothing really concrete*.*”* [C: daughter, 42 years, Milan]. *“It’s an extra for them [the dyads] and also a reason for frustration*, *because they still want support*, *they want help*, *but in fact*, *apart from mediating relations with local health providers*, *offices and so on*, *we couldn’t do anything else for them*.*"* [Female psychologist, Rome]. *“Clearly our role was not one of direct care*, *it was facilitating[…] the picture was clear*. *The problem was how to do that in practice*, *how to accomplish [things]*.*"* [Female psychologist, Milan]. *“l had difficulties understanding what I had to do*, *what I could do*, *and was right to do according to study objectives*.*”* [Female neurologist, Milan].

With regards to the teams, team members reported the following barriers: lack of other health care professionals; need for more teambuilding; insufficient supervision of teams. Caregivers also complained of a lack of other health care professionals on the team, particularly a physiotherapist and speech therapist. *“I’d recommend having a physiotherapist and a speech therapist on the teams*, *to assess specific problems and provide advice*.*”* [C: brother, 48 years, Rome].

Palliative care nurses of the Milan and Rome teams noted the challenge of gaining experience in caring for people with MS. *“Team members had specific competences*, *but some like me lacked skills in caring for MS patients*. *I had to learn about this new disease [*…*] to this was added the need to become familiar with other team members*.*”* [Female nurse, Rome]

All teams reported difficulties with team working and asked for more supervision. “*We didn’t know each other*, *we didn’t have the prerequisites to work as a team in the sense that none of us*, *except for [the team nurse] had team-work experience*.*”* [Female physician, Milan]. *“Working as a team*, *as a group*, *was difficult*. *I’m not sure whether we as a group needed more training or whether this need was more general*, *and regarded how to manage the dynamics that usually manifested within a group*.*”* [Female social worker, Catania].*“Supervision [which was insufficient] is crucial in this kind of job*!*”* [Female psychologist, Milan].

Difficulties of dyad functioning were also recognized as a barrier. Patients and carers had difficulties articulating their needs. *"They asked us many explicit questions but many questions remained unvoiced*: *they came out by asking us for help on issues that were apparently organizational*, *but in fact were about chronic conflicts between family members that impacted on patient care and organization*. *These occurrences were taxing for us*, *since after recognizing these unexpressed questions*, *it was then up to us to figure out whether there were intervention areas or not*.*"* [Female social worker, Catania]. *“What impressed me most was the difficulty that dyads had exploring their needs*, *as if they were not used to doing this*. *I guess the first time they really approached this was when they had the baseline visit*. *For relatives it was too difficult for them to express their personal needs*, *as all their needs were linked in some way to the needs of the patient*. *This required time and work*.*"* [Female psychologist, Milan]. *"[…] for years [problems] are kept there [repressed]*. *You have no access*, *they have no access*, *so even if you realize from certain dynamics there are problems*, *they [the dyads] won’t acknowledge this*.*"* [Female psychologist, Rome].

#### Focus group meetings of referring physicians

The facilitator had great difficulty in placing the comments of the referring physicians in context: they tended to be theoretical and general rather than experiential; they emphasized the challenges of caring for these patients, most of whom had complex symptoms and comorbidities, and distressed and often dysfunctional families: ‘*There are two main problems*: *huge bureaucracy*, *and difficult relations within the families*’ [Family doctor, Rome]. They also noted that MS centres were unsuitable for managing this patient population: ‘*The point is that when the disease progresses the MS centre can offer so little to these patients* …*’* [Neurologist, Rome]. Family doctors pointed out that they followed many patients with chronic diseases, but only a few with severe MS: *‘I am a so called maximalist*, *as I have 1600 patients on charge*, *many with chronic diseases and comorbidities*. *But only five have severe MS’* [Family doctor, Rome]. Referring physicians from Milan were concerned about the unrealistic patient-caregiver expectations that the intervention elicited, and after it closed they would be left with these ‘expectant’ patients. Most physicians said they received no intervention feedback from their patients. Referring physicians from Rome noted that they had not been involved by the teams, but were careful to add that they could have done little due to existing workload: ‘*Well*, *even if they* [the PeNSAMI team] *contacted me*, *I receive so many emails*, *and calls […] In case they required a prescription*, *as an example of a liquid gel thickener*, *I did it*.*’* [Family doctor, Rome]. When asked how the intervention could be improved, the main suggestions were inclusion of a physiotherapist on the team, and replacing the team with a case manager: *‘More than a team*, *they need someone who can collect their needs*, *a case manager*, *not necessarily an health professional*, *who is in close contact with the family*’ [Neurologist, Milan].

## Discussion

This study explored the expectations, the met and unmet needs, and the barriers to intervention success as experienced by PeNSAMI trial recipients and providers. Despite being skeptical, the patient-caregiver dyads had a positive attitude towards the trial, expecting it to increase understanding of people with severe MS and improve disease management. Most of the met needs regarded things that could be directly managed by the teams, with minimal involvement of health/social services, and consisted of improvements in symptom management, psychological wellbeing (due to perception of being supported, reassured, and advised) and some organizational aspects. Nevertheless, some needs remained unmet in each of these categories. Furthermore, barriers, particularly lack of professional support services (chiefly rehabilitation) and networking facilities, and insufficient intervention duration, reduced the effectiveness of the intervention on patients’ and caregivers’ outcomes.

In the PeNSAMI RCT, the HPA teams identified the pre-specified care needs (11 pre-set categories forming three domains, namely “managing everyday life”, “organization”, “psychosocial”) and those which were/were not fulfilled at the end of the intervention [11]. While these three macro areas were also detected in the present qualitative study, the overlap between the sub-categories of needs and their fulfilment is not complete in the two sets of data. Coherently with the quantitative findings, needs about symptoms management, mobility (partially identified with the subcategories “aids” and “medical devices” in the qualitative study) and information were met, while the needs for qualified professionals and relationship improvement (identified with social integration in the qualitative study) resulted still unmet.

Discrepancy between quantitative and qualitative results were detected for emotional wellbeing. This is probably due to two reasons. First, the qualitative study allowed at best to scrutinize this category, identifying different aspects (i.e. emotional support, reassurance, psychological support, management of family problems, reduction of caregiver burden). Second, the aim of qualitative research is depicting stakeholders’ experience independently from its frequency, while that of the quantitative research is finding general trends. This could also explain differences between some improvements reported in the qualitative study and the absence of statistically significant changes in some of the PeNSAMI RCT outcomes, specifically patient quality of life and caregiver burden. In fact, if the control of pain and other symptoms was achieved relatively quickly, a longer term but variable intensity intervention with emphasis on rehabilitation seems necessary in order to improve patient autonomy and social integration. Furthermore, the teams should have been able to call upon MS rehabilitation services in order to effect improvements. Although team members did successfully intercede in some cases, the paucity of available services, the demanding administrative procedures, and lack of networking facilities served as effective barriers. An additional difficulty arose from the fact that teams’ role in the intervention was to assess dyad needs, and activate pertinent services. This indirect role contrasted with the usual hands on working mode of most team members. Paradoxically, some of the good experiences reported by the dyads (e.g. accompanying the caregiver to local offices and prescribing aids and devices) were instances of deviations in trial protocol by team members.

Team members also reported difficulties in building working relationships with patient referring physicians (neurologists or family doctors). This is consistent with outcomes of the referring physician focus groups, whose participants discussed at a theoretical or general level instead of recounting their experiences of the trial–suggesting that they were at best only superficially involved in it. Notwithstanding this, the HPA teams should probably have dedicated more efforts in building up this connection.

Elaboration on the trial experience as a whole was not a pre-specified objective (our focus was the intervention), however the study design consistently emerged as an additional challenge. Having a blinded examiner was a plus in terms of methodological rigor and trial quality, but was a barrier to interaction with team members. Dyads were also confused about the roles of team members and the examiner, which added considerably to caregiver burden.

In a previous study that investigated patient and caregiver needs, caregivers complained about not having time to themselves and difficulties articulating their needs [6]. It is likely that some caregivers viewed their participation in the PeNSAMI trial as an extra duty rather than an occasion for support and respite. In fact neither the PeNSAMI trial [11] nor the Ne-Pal trial [9] found that the intervention alleviated caregiver burden. By contrast, a home based 3-month trial of specialist palliative care in the UK found some improvements in informal caregiver burden (but no effect on primary outcome in patients) although caregiver burden changes were only available for 20/52 (38%) of caregivers [8].

All team members experienced difficulties in team working and it was generally acknowledged that more supervision of teams was required. Notably, the Milan and Rome nurses with longstanding experience in palliative care, reported that dealing with MS sufferers was a new and demanding experience for them.

### Limitations

Although we attempted to capture the gamut of experiences and perspectives of dyads who received the PeNSAMI intervention, it is possible that some diversity may have been missed. Additionally, the Catania referring physician focus group was not held, and we cannot rule out that more experiential (and productive) discussion would have emerged from this meeting. Moreover, while every effort was made to arrange a convenient time for the HPA team focus group, not all team members could attend. It is thus possible that we did not capture some important aspects of team experience. Finally, another important point is the qualitative design of this study that does not allow us to generalize findings beyond this sample. Trial recipients and providers’ narratives should be interpreted as reconstructions of their experiences, rather than the absolute truth.

## Conclusions

This qualitative study embedded in the PeNSAMI trial scrutinized the experiences of recipients and providers of a complex intervention. The findings illuminate the quantitative findings of the trial, and regard three main aspects. The first (in conformity with the aim of the qualitative study) is that the main strengths and limitations of the intervention were identified. Some of the limitations may be overcome by increasing intervention duration, and by investing more time and resources in building a functioning team, well-connected with key services, chiefly MS rehabilitation services. The second aspect is that the facilitating aim of the intervention was to a considerable extent frustrated by the lack of available services. The third aspect pertains to conducting high-quality randomized controlled trials on complex interventions [21]: our effort to obtain internally valid and unbiased findings was worthwhile, but resulted in extra burdens on patients and especially caregivers. This clearly emerged from the experiences of all parties, and points to a need to find new ways to combine the requirements of the experimental method with clinical obligations and patient-centered care [22].

## Supporting information

S1 Checklist Consolidated Criteria for Reporting Qualitative Studies (COREQ) checklist. Completed checklist (except items 30–32) of Consolidated Criteria for Reporting Qualitative Studies (COREQ) to provide supplementary information and locate key points in paper. N.R. is not reported.
(DOCX)

S1 Appendix The PeNSAMI trial: Inclusion criteria and intervention description. (DOCX)

S2 Appendix Interview and focus group meeting guides: Process and developed versions. (DOCX)

S3 Appendix Audit trail. (DOCX)
