# Supplementary material for: Participant perspectives of a home-based palliative approach for people with severe multiple sclerosis: A qualitative study
Source: PLoS One. 2018 Jul 12;13(7):e0200532. doi: 10.1371/journal.pone.0200532 (PMC6042757; doi:10.1371/journal.pone.0200532)
Supplement: S1 Checklist — Completed checklist (except items 30–32) of Consolidated Criteria for Reporting Qualitative Studies (COREQ) to provide supplementary information and locate key points in paper. N.R. is not reported. (DOCX) [file pone.0200532.s001.docx]

| **S1 Checklist. Consolidated Criteria for Reporting Qualitative Studies (COREQ) checklist**  HPA is home palliative approach; N.R. is not reported. | | | |
| --- | --- | --- | --- |
| **1. Research team and reflexivity** | | | |
| Personal Characteristics | Description/Paper quotation | Where in paper | |
| 1. Interviewer/facilitator | *Interviews - ‘*The interviews were conducted in Milan by a psychologist experienced in qualitative research (EB), and in Rome and Catania by specially trained psychologists (SCip and SC, respectively).’  *Focus groups* - ‘All were conducted by a single facilitator (EB, not acquainted with any participant except the psychologist of the team of Milan) […] The co-moderator (AS) took notes and oversaw the audio recording.’ | Methods  Methods | |
| 2. Credentials | MSc, psychologists | N.R. | |
| 3. Occupation | CB was the head of the Unit of Clinical Psychology, Foundation IRCCS Istituto Nazionale per la Cura dei Tumori, Milan Italy). EB was a researcher at the same Unit. SC and SCip were freelance clinical psychologists. |  | |
| 4. Gender | Female interviewers/facilitator | N.R. | |
| 5. Experience & training | CB, EB had longstanding experience in qualitative research. SC and SCip were trained by CB and EB for the purpose of this study. | N.R. | |
| Relationship with participants | | | |
| 6. Relationship established | *Interviews* - ‘Neither patients nor carers had met the interviewers previously.’  *Focus groups* - ‘All were conducted by a single facilitator (EB, not acquainted with any participant except the psychologist of the team of Milan)’ | Methods  Methods | |
| 7. Participant knowledge of the interviewer | No knowledge. | N.R. | |
| 8. Interviewer characteristics | Interviewers and the facilitator were part of the Qualitative Analysis Panel (see Acknowledgements). They were specifically concerned with interviews and focus group meetings and did not participate in trial activities. | Acknowledgements | |
|  | | | |
| **2. Study design** | | | |
| Theoretical framework | | | |
| 9. Methodological orientation & theory | ‘The methods of framework analysis were applied to the data.’ | Methods | |
| Participant selection | | | |
| 10. Sampling | *Interviews* - ‘A purposive sampling technique was used to select the participants (at least four dyads from each center) who obtained the greatest and least benefit from the intervention (in terms of primary outcomes). At least two caregivers of patients with severe cognitive compromise (patients not interviewed) were also selected.’  *Focus groups* - All referring physicians of the dyads who received the HPA intervention, and all HPA team members, were invited to take part in the focus group meetings. | Methods  N.R. | |
| 11. Method of approach | *Interviews* - Eligible dyads were first identified by the coordinating unit (see sampling above). They were then approached by the center PI or a HPA team member, who informed them of study aims and procedures, and asked them to provide informed consent to participate.  Consenting dyads were then contacted by the interviewer, who further detailed the interview procedure and scheduled the interview. Before the interview, the dyads/caregivers signed in the informed consent.  *Focus groups* – Referring physicians of the dyads who received the intervention were approached by the trial or the center PI, who informed them of study aims and procedures, and asked them to provide informed consent. HPA team members were approached by the study PI (AS) or by the center PI. | N.R.  N.R. | |
| 12. Sample size | *Interviews* - ‘27 interviews of mean duration 28 minutes (range 11–60) were conducted, 12 with patients and 15 with caregivers (Table 1).’  *Referring physician focus groups* - ‘The characteristics of the eight participants (4 per meeting) are shown in Table 2.’  *HPA team focus group -* ‘6 participated in person, two via videoconference (GO, Catania team social worker; AMG, Milan psychologist) and one (MCS, Catania nurse) via audio-conference.’ | Results  Results, Table 2 | |
| 13. Non-participation | *Interviews* - ‘Two of the contacted dyads refused to participate: one because unavailable during the period allocated for interviews, the other because the carer was too occupied with caregiving.’  *Referring physician focus groups* - ‘Of the 34 eligible physicians, 21 (61.7%) refused to participate. Reasons for refusal were work commitments (n=19) and holidays (n=2). Five agreed to participate but did not show up for unscheduled work commitment (n=3), family commitment (n=1), and oversight (n=1).’  *HPA team focus group -* ‘Three could not attend, 2 for work commitments (physician and social worker of Rome team) and the other for personal reasons (Catania team psychologist).’ | Results  Results | |
| Setting | | | |
| 14. Setting of data collection | *Interviews* - ‘Patients and caregivers were interviewed separately at patient homes.’  *Focus groups* - ‘The referring physician focus group meetings took place in June (Besta Neurological Institute, Milan, 60 minutes) and July (S. Lucia Rehabilitation Hospital, Rome, 90 minutes) 2016’.  ‘The HPA team meeting was held at the Besta Neurological Institute, Milan, in October 2016 and lasted 120 minutes.’ | | Methods  Results |
| 15. Presence of non-participants | *Interviews -* Caregivers were alone with the interviewer. Patients were also alone with the interviewer, but a caregiver (formal or informal) was nearby to assist the patient (to drink, change position etc.).  *Focus groups* - No non-participant was present | | N.R.  N.R. |
| 16. Description of sample | *Interviews* - Table 1  *Focus groups* - Table 2 | | Table 1  Table 2 |
| Data collection | | | |
| 17. Interview guide | ‘Guides to conducting the interviews and focus group meetings (S2 Appendix) were developed by the PeNSAMI qualitative panel, with input from the steering committee. After piloting in two dyads, minor changes were made to the structure and order of the questions in the interview guide.’ | | Methods, S2 Appendix |
| 18. Repeat interviews | No interviews were repeated | | N.R. |
| 19. Audio/visual recording | *Interviews* - ‘Participants were assured that the interviews were confidential, and that the audio recordings and subsequent transcripts would be fully anonymized.’  *Focus groups* - ‘The co-moderator (AS) took notes and oversaw the audio recording.’ | | Methods  Methods |
| 20. Field notes | *Interviews* - ‘The interviewer also noted any potentially informative non-verbal gestures.’  *Focus groups -* ‘The co-moderator (AS) took notes and oversaw the audio recording. Subsequently, the facilitator produced a report from the audio recordings/transcript and her field notes, which was submitted to participants for review (respondent validation).’ | | Methods  Methods |
| 21. Duration | *Interviews* - ‘Between October 2015 and April 2016, 27 interviews of mean duration 28 minutes (range 11–60) were conducted, 12 with patients and 15 with caregivers (Table 1).’  *Referring physician focus groups* - ‘The referring physician focus group meetings took place in June (Besta Neurological Institute, Milan, 60 minutes) and July (S. Lucia Rehabilitation Hospital, Rome, 90 minutes) 2016.’  *HPA teams focus group -* ‘The HPA team meeting was held at the Besta Neurological Institute, Milan, in October 2016 and lasted 120 minutes.’ | | Results  Results  Results |
| 22. Data saturation | ‘A minimum of 12 patient and 12 caregiver interviews was planned. Data from each set of interviews were analyzed immediately and used to decide the characteristics of the next interviewee dyad, revise the interview guide (if necessary), and indicate when interviewing should cease because of data saturation [14].’ | | Methods |
| 23. Transcripts returned | *Interviews* – NO  *Focus groups* - ‘Subsequently, the facilitator produced a report from the audio recordings/transcript and her field notes, which was submitted to participants for review (respondent validation).’ | | Methods |
|  | | | |
| **3. Analysis and findings** | | | |
| Data analysis | | | |
| 24. Number of data coders | ‘Two psychologists (EB and CB) experienced in qualitative research and not involved in MS patient care, analyzed the transcripts […].’ | | Methods |
| 25. Description of the coding tree | ‘Two psychologists (EB and CB) experienced in qualitative research and not involved in MS patient care, analyzed the transcripts in six steps, independently (steps 1-5) and jointly (step 6): (1) In a given transcript the researcher identified all propositions thought significant, without considering their relation to other parts of the transcript, and added comments. (2) Comments were subsequently expanded and related to other points that arose. (3) Relations between comments were established by re-ordering and re-grouping by sub-categories. (4) The sub-categories considered relevant were ordered hierarchically into categories, moving from the specific to the general. (5) The analyses of each transcript were then compared with each other to identify common and one-off sub-categories. (6) The analyses produced by each researcher were compared, and a consensus arrived at. The six phases of the analysis are exemplified in S3 Appendix.’ | | Methods, S3 Appendix |
| 26. Derivation of themes | ‘A joint report of interview and focus group meeting findings was produced. In this report − and the present paper − the categories identified were compared with those identified in the qualitative study used to inform the development of the PeNSAMI intervention [6]. Where appropriate, the previous category names were used to facilitate comparison.’ | | Methods |
| 27. Software | Not used. | | N.R. |
| 28. Participant checking | *Interviews* - ‘At the end of the interview, the interviewer verbally summarized the key points and asked the participant if the summary was full and correct.’  *Focus groups* - ‘After all pre-specified topics had been fully discussed, the facilitator summarized the main points, and asked for further feedback and whether all concerns had been fully aired. The co-moderator (AS) took notes and oversaw the audio recording. Subsequently, the facilitator produced a report from the audio recordings/transcript and her field notes, which was submitted to participants for review (respondent validation)’. | | Methods  Methods |
| Reporting | | | |
| 29. Quotations presented | Yes | | Results |
| 30. Data & findings consistent | – | |  |
| 31. Clarity of major themes | – | |  |
| 32. Clarity of minor themes | – | |  |
