# Supplementary material for: Participant perspectives of a home-based palliative approach for people with severe multiple sclerosis: A qualitative study
Source: PLoS One. 2018 Jul 12;13(7):e0200532. doi: 10.1371/journal.pone.0200532 (PMC6042757; doi:10.1371/journal.pone.0200532)
Supplement: S1 Appendix — (DOCX) [file pone.0200532.s002.docx]

**S1 Appendix. The PeNSAMI trial: Inclusion criteria and intervention description**

The PeNSAMI trial was a multicentre phase II/III single-blind randomized controlled trial. Participants (dyads of adults with severe multiple sclerosis (MS) and their carers) were randomized to either six-month home-based palliative care approach (HPA) or usual care. The trial was registered with Current Controlled Trials (ISRCTN73082124). The study protocol was published [10].

*Inclusion criteria*

All adults (18 years or older) who fulfilled all the following criteria were potentially eligible:

1. Diagnosis of MS (Polman 2011 criteria);
2. Expanded Disability Status Scale (EDSS) ≥8.0 [21];
3. Primary or secondary progressive course;
4. Presence of a carer: family member, relative, or friend, who was next of kin or key decision maker as designated by the (cognitively competent) MS patient and with whom the patient shared his/her life;
5. At least two unmet care needs among the categories identified in the PeNSAMI qualitative study preceding the trial (Table) [6], or the patient declared for comfort care only;
6. One or more of the following: significant complex symptoms/medical complications, dysphagia/poor nutritional status, communication difficulties (http://www.goldstandards framework.org.uk).

*Exclusion criteria* were:

1. Hospitalized/institutionalized patients;
2. Patients already receiving palliative care;
3. Dyads living out of study area.

**Table.** List of main care need categories, as identified in the PeNSAMI Phase 1 qualitative study

| **Domain** | **Category** |
| --- | --- |
| ‘Managing everyday life’ | Symptoms management  Personal care/hygiene  Activities of daily living  Outdoor mobility and transport |
| ‘Psychosocial’ | Relationships/communication  Leisure/holidays  Psychological well-being/social role |
| ‘Organization’ Information | Access to services  Co-ordination of services  Competent professionals |

*Intervention*

The HPA was based on the principles of palliative care as described in the 2010 guidance document of the UK National Council for Palliative Care, Neurological Alliance, and National End of Life Care Programme. Each centre had a team consisting of a neurologist, a nurse (case manager and team leader), a psychologist, and a social worker. The nurses of the centres of Milan and Rome had degrees in palliative care; the Catania nurse attended a week-long individual training course. All team members were trained in the intervention. Initially, the team formed a comprehensive assessment of each dyad. The intervention content for each dyad was then developed, discussed with the dyad and the patient caring physician, and delivered over six months, with involvement of local services (the intervention was not intended to replace existing services but to complement and enhance them). Subsequently, the team verified program implementation, and reviewed/modified it as necessary. Home visits by one or more team members took place at least twice a month in the first trimester, and as needed thereafter; which professionals attended for home visits depended on the intensity of care and the type of symptoms. A dedicated phone number was given to dyads to enable easy communication with the team. The team was not on call for patients: in the event of emergencies, dyads contact the caring physician or emergency medical services.
