# Supplementary material for: Participant perspectives of a home-based palliative approach for people with severe multiple sclerosis: A qualitative study
Source: PLoS One. 2018 Jul 12;13(7):e0200532. doi: 10.1371/journal.pone.0200532 (PMC6042757; doi:10.1371/journal.pone.0200532)
Supplement: S2 Appendix — (DOCX) [file pone.0200532.s003.docx]

**S2 Appendix. Interview and focus group meeting guides: process and developed versions**

**Method of guides construction**

For the Interview construction we used the four-step method of inquiry described in McCracken G. *The Long interview.* London: Sage, 1998.

The interview construction began with an exhaustive **review of the literature** that enable investigators to establish the domain the interview will explore and to define the object of the study.

The second step consisted in the **review of cultural categories** where the investigators account and examine the associations, incidents and assumptions that surround the topic that have not been considered by the literature.

The third step regarded the **questionnaire construction and Interview procedure.**

The fourth step focused on the **discovery of analytical categories.**

**Timeline:**

**July 2015**

The members of the PeNSAMI Qualitative Analysis Panel produced the PeNSAMI First Interview guide (page 2).

**October 2015**

The interview guide was piloted with the dyads #01-05; 01-07 which were analyzed. Based on this analysis the guide was reviewed by adding the following four questions: 1) Going back to the beginning of the trial, how did you feel about the proposal to participate? What did you expect from the trial? 2) At the end of the trial, which of your needs were still unmet? What are your needs at present? 3) Did any new goals emerge during the intervention? 4) Do you have any suggestions for the team, and for the PeNSAMI trial as a whole? Please give your suggestions. The PeNSAMI final interview guide was then finalized (page 3).

**22^nd^ October 2015**

The first interview was performed. The interview guide for caregivers of patients cognitively compromised was created. The same questions were administered to the caregivers asking about his/her perspective and that of his/her relative.

**May 2016**

The PeNSAMI patient referring physicians focus group guide was finalized and no further modifications were made (page 4).

**August/September 2016**

FGMs analysis and decision to run a FGM with the PCT. The FGM guide for PCT was finalized and no further modifications were made. See PeNSAMI teams focus group guide (page 5).

**PeNSAMI First Interview guide**

1. EXPECTATIONS

- Please describe your experience with the PeNSAMI team.

1. GOAL SETTING AND ACHIEVEMENT

- What were the main needs you had? Did you express these needs to the team?
- What were your goals from the intervention? Did the team discuss these goals together with you?
- Which of your needs were met in the six months of the intervention?
- Did any new needs emerge during the intervention? Were these new needs discussed with the team?

1. SATISFACTION WITH THE INTERVENTION

- Are you satisfied with the intervention?
  - Are you satisfied with the way the intervention was conducted?
  - Are you satisfied with the timetabling and duration of the intervention?
  - Are you satisfied with the individual members of the PeNSAMI team (their professionalism and expertise) and with the team as a whole?

1. PARTICIPANT SUGGESTIONS/ FUTURE

- Did you expect anything different?
- Are there things you would change? What would you change?
- Would you have liked to continue being followed by the PeNSAMI team?
- What are your needs today?
- Do you think that the PeNSAMI team would be able to satisfy these needs?

**PeNSAMI final interview guide**

1. EXPECTATIONS

- Please describe your experience with the PeNSAMI team.
- Going back to the beginning of the trial, how did you feel about the proposal to participate? What did you expect from the trial?

1. GOAL SETTING AND ACHIEVEMENT

- What were the main needs you had? Did you express these needs to the team?
- What were your goals from the intervention? Did the team discuss these goals together with you?
- Which of your needs were met in the six months of the intervention?
- At the end of the trial, which of your needs were still unmet? What are your needs at present?
- Did any new needs emerge during the intervention? Were these new needs discussed with the team?
- Did any new goals emerge during the intervention?

1. SATISFACTION WITH THE INTERVENTION

- Are you satisfied with the intervention?
  - Are you satisfied with the way the intervention was conducted?
  - Are you satisfied with the timetabling and duration of the intervention?
  - Are you satisfied with the individual members of the PeNSAMI team (their professionalism and expertise) and with the team as a whole?

1. PARTICIPANT SUGGESTIONS/ FUTURE

- Did you expect anything different?
- Are there things you would change? What would you change?
- Do you have any suggestions for the team, and for the PeNSAMI trial as a whole? Please give your suggestions.
- Would you have liked to continue being followed by the PeNSAMI team?
- What are your needs today?
- Do you think that the PeNSAMI team would be able to satisfy these needs?

**Patient referring physician focus group guide**

1. EXPECTATIONS

- What were your feelings about the PeNSAMI trial when you were asked to participate?
  - How did you welcome the invitation to participate?
  - What did you expect from this experience?

1. INVOLVEMENT IN THE TRIAL

- Did you feel involved in the intervention?
- Did you receive any feedback from your patient or carer about their experience with the intervention? Did any feedback points strike or surprise you?
- Did you notice any changes in your patients during the intervention? What changes?

1. SATISFACTION WITH THE INTERVENTION

- Based on your experience, what do you think about the PeNSAMI trial?
  - Are you satisfied with the care pathway, timetabling, and activities and personnel of the team?
  - Did you notice any effect of the trial (positive or negative) on your working activities? If yes, what effects?
- Did the PeNSAMI trial meet any needs of your patients? What needs?

1. SUGGESTIONS/FUTURE

- Based on your experience, what could have improved the intervention?
- Do you have any suggestions for the PeNSAMI team? Please give your suggestions.
- Would you have liked your patient(s) to continue with the intervention?

**PeNSAMI teams focus group guide**

1. EXPECTATIONS

- What were your feelings about the PeNSAMI trial when you were asked to participate (as a team member)?
  - Did you welcome the proposal?
  - What did you expect from the experience of participating?

1. ACTIVITIES

- What was the training like?
- What was your team experience like?
  - Regarding your work in patient homes? Did you feel personally involved in the experiences of the dyads?
  - What was the team work like?
  - What was the intervention planning like?
  - Were the timetabling and conduct of the visits appropriate?
  - What was your impression of the team meetings?
  - What was your impression of the supervision?
  - What was your impression of interactions with the patient referring physicians?
- Did you find any critical issues? (note: if anybody mentions the blind examiner, ask about it)

1. DYAD NEEDS

- What were the main needs expressed by dyads?
- Did you notice any mismatch between expressed needs and those you observed directly?

1. INTERVENTION EFFECTS

- Based on your experience, what is your opinion about this trial?
- Do you think you team met the dyad needs?
  - Did you receive any feedback from patients or carers?
  - Did you notice any changes in the dyads?
  - Did you perceive any specific effects on your working activities? If yes, what effects?
- Would you have liked dyads to continue with the intervention?
- Do you have any suggestions for intervention improvement/revision?
- Based on your experience, do you think that the care model (embodied in the intervention) is applicable?
