# Supplementary material for: Participant perspectives of a home-based palliative approach for people with severe multiple sclerosis: A qualitative study
Source: PLoS One. 2018 Jul 12;13(7):e0200532. doi: 10.1371/journal.pone.0200532 (PMC6042757; doi:10.1371/journal.pone.0200532)
Supplement: S3 Appendix — (DOCX) [file pone.0200532.s004.docx]

**S1 Appendix. Audit trail – Qualitative analysis PeNSAMI study**

**Index**

- Timeline p. 2-3
- The six steps of the analysis p. 4-7

**Timeline**

**July 2015**

EB and CB developed the personal individual interview (PSI) grid draft. They shared these first versions with the other members of the Qualitative Analysis Panel (QAP): E Bianchi; S Cilia: University Hospital Policlinico Vittorio Emanuele, Catania; S Cipollari: IRCCS S Lucia Foundation, Rome; E Pietrolongo, M Giuntoli, and C Borreani.

Members of the QAP took decisions about how selecting and contacting dyads for the qualitative study. In particular, QAP decided: 1) not to involve the intervention teams in the selection of dyads to interview, in order not to be influenced by their opinion; 2) the intervention teams sent the dyads the clinical report of the intervention after the dyads were interviewed, as to prevent any bias.

**September 2015**

The PSI gird was finalized by enriching them with prompts to facilitates dyads answer in case of impasse.

**September/October 2015**

Dyads form Milan (Coordinating centre) were selected and invited to participate in the interviews.

**22^nd^ October 2015**

The first interview was conducted at dyad home.

After this first interview the PSI grid was reviewed and the one regarding patients cognitively compromised was developed.

In addition, the dyad selecting procedure was modified: the QAP decided to ask intervention teams to provide information about dyads ability of being informative.

**January/February 2016**

EB and CB performed the first qualitative analysis on a subsample of 9 interviews (4 patients and 5 caregivers).

**15^th^ February 2016**

Meeting with the intervention teams to provide them with feedbacks from the analysis run so far and discussion with them about these preliminary results.

**April/May 2016**

EB and CB selected the last dyads according to the purposive sampling criteria.

**May 2016**

EB and CB developed the grid draft for the FGM with patient referring physicians.

**7^th^ June 2016**

EB and AS run the FGM with patient referring physicians in Milan. A lay summary of the FGM was validate by each participant.

**26^th^ July 2016**

EB and AS run the FGM with patient referring physicians in Rome. A lay summary of the FGM was validate by each participant.

**August/October 2016**

EB and CB analysed FGM transcripts. A lay summary of the FGM was validate by each participant.

As experiential data were negligible from analysis of two referring physician meetings, the QAP decided not to hold the third. An additional FGM involving all the three intervention teams was also planned (not contemplated in study protocol).

The FGM grid with the intervention teams was developed by September 2016 and the FGM run the 14^th^ October 2016 in Milan.

**October/November 2016**

EB and CB performed the joint analysis of PSIs and FGMs.

**The six steps of the analysis**

1. In a given transcript the researcher identified all propositions thought significant, without considering their relation to other parts of the transcript, and added comments.
   Emerging topics from PSIs: an example by EB.

| ***Argomenti identificati*** |
| --- |
| *Curiosità per il progetto*  *Speranza per maggiore aiuto pratico*  *Trasmissione di di informazioni*  *Visite mediche a domicilio*  *Avere dei riferimenti costanti e formati, competenti e disponibili*  *Migliorare l’attivazione della fisioterapia e della logopedia*  *Avere un aiuto psicologico costante per i pazienti (anche al domicilio)*  *Sollevare il caregiver*  *Vedere qualcuno / compagnia / poter comunicare e parlare di sè*  *Creazione di un punto di riferimento*  *Possibilità di confronto*  *Avere un riferimento costante*  *Uscire (avere persone e mezzi disponibili per gli spostamenti)*  *Percezione di attenzione, gentilezza e discrezione degli operatori*  *Interesse “umano”*  *Speranza di un miglioramento della malattia*  *Interventi poco numerosi*  *sempre la presenza del caregiver*  *Difficoltà nel ricevere in casa estranei*  *Test dell’esaminatore in cieco vissuti come lunghi e poco centrati sulla persona*  *Sorpresa per la novità*  *Fiducia nei neurologi curanti per la proposta*  *Poter ricevere consigli e informazioni*  *Potersi confrontare con altri “esperti”*  *Potersi sfogare con qualcuno*  *Scetticismo, nessuna attesa di cambiamento positivo*  *Attesa di qualcuno che vede come funzionano le cose*  *Pratiche burocratiche*  *Mancanza del fisioterapista e logopedista in équipe*  *Interventi solo abbozzati per mancanza di tempo*  *Gli interventi richiedevano Adeguamento ausili (sedia a rotelle, pannoloni) o infrastrutture (ascensore, parcheggio)*  *Monitoraggio e gestione dei sintomi (dolore, spasticità, piaghe) e procedure mediche (PEG)*  *Sostegno psicologico/attivazione supporto psicologico da risorse territoriali*  *Sensazione di non essere più soli*  *Sensazione di cura, rassicurazione*  *Avere un punto di riferimento, qualcuno su cui contare*  *Avere un ponte tra malato e istituzioni*  *Maggiori relazioni: “Qualche faccia nuova”* |

1. Comments were subsequently expanded and related to other points that arose.
   An example of integration between patients and caregivers topics.

| ***Pazienti*** | ***Caregivers*** |
| --- | --- |
| *Scetticismo*  *Miglioramento della malattia*  *Qualcosa di utile*  *Curiosità*  *Qualcuno che si vuole informare sulla condizione*  *Poche aspettative “disincantate”* | *Aumentare conoscenze*  *Miglioramento della malattia*  *Ricevere consigli*  *Ricevere la visita di qualcuno in generale / di persone competenti*  *Ricevere un intervento pratico / concreto*  *Nessun cambiamento drastico*  *Poter parlare della propria esperienza / farla conoscere per migliorare la ricerca* |
| *Migliorare i sintomi*  *Ricevere cure mediche*  *Parlare con i medici*  *Dare un sostegno al caregiver*  *Vedere persone nuove, avere compagnia*  *Poter uscire* | *Avere consigli / informazioni*  *Avere un riferimento costante*  *Ricevere visite mediche al domicilio*  *Bisogno di “staccare”*  *Migliorare i servizi socio-sanitari*  *Essere tenuti in considerazione* |
| *Informazioni e consigli*  *Attenzioni e interesse per sè*  *Interventi burocratici (ausili)*  *Diminuzione dolore e miglioramento altri sintomi*  *Potersi relazionare con qualcuno di nuovo*  *Essere ascoltati*  *Potersi confrontare ed esprimere* | *Aggiornamenti*  *Spiegazioni*  *Informazioni*  *Sicurezza*  *Confronto*  *Sfogo delle frustrazioni/aiuto morale*  *Miglioramento ausili e sintomi del malato*  *Attivazione di procedure mediche (PEG, visite)* |
| *Parlare con i medici*  *Fisioterapia*  *Compagnia*  *Essere di aiuto / Sentirsi utili*  *Poter uscire di più / socializzare*  *Sostegno psicologico*  *Miglioramenti architettonici*  *Avere persone di aiuto e competenti* | *Coordinamento tra i servizi*  *Essere aggiornati sempre*  *Avere riferimenti competenti*  *Migliorare e accelerare le pratiche*  *Sostegno psicologico*  *Avere aiuto nella gestione pratica e organizzativa*  *Potersi occupare della propria salute* |
| *Gentilezza*  *Interesse da parte dell’equipè*  *Poter dialogare con qualcuno di competente*  *Sensazione che ci fosse un coordinamento*  *Constatare l’effettivo interessamento*  *Vedere qualcuno che si impegna / che ha cura* | *Interessamento / Attenzioni ricevute*  *Essere stati capiti*  *Punto di riferimento*  *Rassicurazione*  *Sentirsi protetti*  *Sentirsi meno soli*  *Umanità degli operatori* |
| *Confusione circa le figure intervenute*  *Troppe domande in alcuni interventi*  *Necessario il coinvolgimento famigliare non sempre possibile*  *Illusione che si potesse fare qualcosa di più* | *Pochi risultati nella pratica, mancanza di rete*  *Doversi ripetere a persone diverse*  *Troppe domande in alcuni interventi*  *Poco chiaro chi fossero i vari operatori*  *Necessari più incontri*  *Mancava fisioterapista in equipe*  *Senso di intrusione*  *Interventi abbozzati per mancanza di tempo*  *Illusione che si potesse fare qualcosa di più* |

1. Relations between comments were established by re-ordering and re-grouping by sub-categories.

The following table includes all the sub-categories identified, showing differences and communalities between the two researchers. N/R is note reported.

| ***EB*** | ***CB*** |
| --- | --- |
| - *N/R* - *Disincanto* - *Gestione della malattia* - *Condivisione dell’esperienza di malattia* - *Avere l’opinione degli esperti* - *Avere supporto concreto* - *Miglioramento clinico* - *Migliorare la conoscenza scientifica* - *Gestione dei sintomi* - *Ausili* - *Punto di riferimento* - *Cure domiciliari* - *Professionisti sanitari e case manager competenti* - *Fisioterapia* - *Supporto emotivo* - *Rassicurazione* - *Comunicazione* - *Informazione* - *Aspetti amministrativi* - *Integrazione sociale* - *Essere utile* - *Supporto psicologico* - *Gestione dei problem familiari* - *Riduzione del carico assistenziale del caregiver* - *Servizi insufficienti* - *Mancata integrazione tra i servizi* - *Procedure amministrative complesse* - *Abitazioni inadeguate* - *Intervento troppo breve* - *Carico dovuto alle visite dell’esaminatore e alle telefonate mensili* - *Difficoltà delle diadi a identificare il ruolo dell’esaminatore e del team* - *N/R* - *Azione indiretta del team* - *Mancanza di altri professionisti sanitari* - *Insufficiente teambuilding* - *Insufficiente supervisione dei team* - *Difficoltà nell’espressione dei bisogni* - *Diadi disfunzionale* | - *Curiosità* - *Scetticismo* - *Gestione della sclerosi multipla* - *Condivisione dell’esperienza di malattia* - *Avere l’opinione degli esperti* - *Ricevere supporto* - *Miglioramento nelle condizioni di salute* - *Migliorare la conoscenza scientifica* - *Gestione dei sintomi* - *Presidi* - *Punto di riferimento* - *Assistenza domiciliare* - *Project manager e professionisti dedicati* - *Fisioterapia* - *N/R* - *Rassicurazione* - *Comunicazione* - *Informazione* - *Burocrazia* - *Socializzazione* - *Aiutare gli altri* - *Supporto psicologico* - *Dinamiche familiari* - *Riduzione del carico assistenziale del caregiver* - *Mancanza di servizi* - *Frammentazione dei servizi* - *N/R* - *Abitazioni inadeguate* - *Brevità intervento cure palliative* - *Peso dell’esaminatore in cieco* - *N/R* - *Confusion sul ruolo di team e esaminatore* - *Invadenza* - *Azione indiretta del team* - *Mancanza di altri professionisti sanitari* - *Team poco coeso* - *Più supervisione per i team* - *Difficoltà nell’espressione dei bisogni* - *N/R* |

Some examples of sub-categories and corresponding proposition are provided:

- *Professionisti sanitari e case manager competenti: necessità di un case manager che organizzi le visite*
- *Informazione: consigli utili forniti da infermiere e assistente sociale*
- *Servizi insufficienti: mancanza di visite domiciliari*
- *Difficoltà nell’espressione dei bisogni: pazienti e caregiver hanno difficoltà a riconoscere e esprimere i propri bisogni*

1. The sub-categories considered relevant were ordered hierarchically into categories, moving from the specific to the general. See the following table as an example of EB analysis.

| ***Categoria*** | ***Organizzazione*** |
| --- | --- |
| *Sottocategorie* | *Procedure amministrative complesse*  *Mancata integrazione tra i servizi* |

1. The analyses of each transcript were then compared with each other to identify common and one-off sub-categories. See the following table as an example of the addition of one sub-category, after the comparison of the analysis of different transcripts (i.e. “servizi sufficienti” was added) from EB analysis.

| ***Categoria*** | ***Organizzazione*** |
| --- | --- |
| *Sottocategorie* | *Procedure amministrative complesse*  *Mancata integrazione tra i Servizi*  *Servizi insufficienti* |

1. The analyses produced by each researcher were compared, and a consensus arrived at. The results of this consensus analysis are reported in tables 4,5,6 of the manuscript.
